# Estimates of COVID-19 Risk Factors among Social Strata and Predictors for a Vulnerability to the Infection

**DOI:** 10.3390/ijerph18168701

**Published:** 2021-08-18

**Authors:** Dimitra S. Mouliou, Ourania S. Kotsiou, Konstantinos I. Gourgoulianis

**Affiliations:** Department of Respiratory Medicine, Faculty of Medicine, University of Thessaly, BIOPOLIS, 41110 Larissa, Greece; raniakotsiou@gmail.com (O.S.K.); kgourg@uth.gr (K.I.G.)

**Keywords:** surveillance, COVID-19, symptoms, vulnerability, generation, economy, sectors

## Abstract

Coronavirus disease 2019 (COVID-19) has emerged as a potentially severe disease, especially for individuals presenting with certain underlying medical conditions. We analyzed the rates of comorbidities and symptoms to reveal the potential severity of the pandemic in Volos, one of the most air-polluted cities in Greece. Environmental and health-related predictors for SARS-CoV-2 infection were investigated. A web-based questionnaire was disseminated through social media in the first half of March 2021 during a five-month strict lockdown. Sociodemographic data, preexisting medical conditions, frequency of clinical symptoms, and COVID-19 information were recorded. The study population consisted of 2000 responders. Four-fifths of the participants reported comorbidities that could increase vulnerability to severe COVID-19. Respiratory symptoms were reported from the unemployed and from retirees, and cold-related symptoms were reported in the education sector and in undergraduates. Women and younger generations shaped social vulnerability to respiratory infections similar to the elderly. SARS-CoV-2 infection was reported in 3.7% of the study population. Common headache (OR 2; CI 1189–3013; *p* = 0.007) and prior pneumonia (OR 1.9; CI 1024–2898; *p* = 0.04) were significant predictors for susceptibility to SARS-CoV-2 infection. The importance of monitoring society through community-based questionnaires is highlighted, for predicting and preventing future widespread transmission of infectious diseases.

## 1. Introduction

Coronaviruses have stigmatized humanity since the beginning of the 21st century. A novel coronavirus disease 2019 (COVID-19) was reported from a cluster of cases of pneumonia in December 2019, in Wuhan, China [1]. Previously, emergency, respiratory, and epidemiological scientific communities have made many attempts to monitor COVID-19. Moreover, risk factors for a possible severe SARS-CoV-2 infection, mainly age and certain underlying medical conditions, have regularly been demonstrated by the Center for Disease Control and Prevention (CDC) [2]. However, viral pathogenesis has been underestimated by numerous pathogenic mechanisms and is influenced by a variety of factors, including environmental, and host-related parameters.

Conversely, the societal prevalence of the reported risk factors for a potential severe COVID-19 infection has shaped the overall pandemic gravity at a societal level, and communities with preexisting medical conditions display weak resistance to further pathogens and social resilience, even if transmission rates are low [3,4]. Vulnerable populations may also present identifiable reasons for their potential vulnerability [5]. It is, therefore, crucial to monitor all possible societal health conditions effectively and identify vulnerable areas for preventive medicine plans to be implemented. Clinical phenotypes from heterogeneous diseases such as COVID-19, with mild to severely symptomatic cases, illustrate the overall susceptibility to inflammation and are dependent on various host, pathogenic, and environmental factors [6]. Therefore, it is critical to detect possible predictors for a rounded susceptibility to SARS-CoV-2 infection.

The latest fourfold increase in infectious diseases and the proven severity of respiratory pandemics are raising awareness of public health safeguards. Airborne transmission marks infectious diseases [7]. COVID-19 is considered a vascular disease, with its main clinical manifestations related to the lungs. Thus, the respiratory profile of society and its vulnerability to infections must be closely monitored to assess the severity of the pandemic and to further evaluate public health maintenance policies. Since urban cities are ground zero sites for viral transmission [8], and environmental risks such as air pollution affect the severity of an infection, it is of fundamental importance that urban communities are monitored, in an age where even urban sound environmental changes are monitored [9]. In particular, since tertiary sector services constitute the lion’s share of the economy, and services also mean the social communications and interactions that are the basis for the transmission of aerosol viruses, this sector must be closely monitored for potential respiratory vulnerability to future pathogens. Therefore, thus far, no literature data reveal such conceptualizations in parallel.

Digitized life has permeated various health-related domains, and social networks have received public attention to a great extent, thus serving as a sophisticated target for monitoring society [10]. The latest epidemiological strategies, shifting toward modernization, are embracing the internet as a pioneering tool for futuristic research, exploiting various terrains, such as the revolution of conventional data collection methods. Although online web-based questionnaires (WBQs) are currently considered a fluid form for studies, they are an increasingly beneficial tool, and, no doubt, they enable experts to combine ontological, ethical, and epistemological principles to beneficially monitor communities. Moreover, WBQs enable motivated individuals to provide their answers rapidly, at a touch of a button, and are automated, cost effective, and error-free [11,12,13]. Therefore, WBQs can be applied to assess the health profile of society, for better screening and control of the specific factors that weaken public health. Moreover, emergency incidents and the severity of infectious diseases can be further prevented by screening social pathology and treating its vulnerable areas.

In this study, in contrast to existing virocentric surveys, an overall evaluation of the potential severity of the COVID-19 pandemic is presented, at an anthropocentric and societal level. A modern epidemiological e-surveillance is manifested, predicated on in toto qualitative e-screening via a WBQ. The frequency rates of risk factors for a possible severe COVID-19 among genders and generations are demonstrated, and economic sectors with a potential vulnerability to respiratory infectious diseases are highlighted, according to the studied respiratory-related symptoms. A rounded investigation of potential predictors for susceptibility to SARS-CoV-2 infection is finally presented.

## 2. Materials and Methods

### 2.1. E-Surveillance Strategy

The survey was designed solely for the first half of March 2021, during an ongoing five-month strict Greek lockdown. From 7 November 2020, only basic movements were allowed to the public via short message service (SMS) authorization, and most tertiary sector services switched to distance work. Greece recorded high daily COVID-19 figures in the first half of March, and the Greek society could be precisely indicated to be in the third wave of direct widespread exposure to the COVID-19 pandemic. Furthermore, especially Volos’s ambient particulate matter (PM) daily figures were higher than their allowed concentrations even during the lockdown—contrary to other European cities where air pollution was less reported due to lockdown policies; therefore, the survey could directly assess air pollution as an active parameter. Moreover, the long-lasting lockdown led to a unique sedentary lifestyle. Likewise, in Greece, some degree of social insouciance in complying with restrictions in late winter was observed. It was important that the age ranges of COVID-19 vaccination began to expand at that point of time in Greece, and thus, it was crucial to reveal the vulnerable populations and economic sectors that would need to obtain vaccination immediately.

### 2.2. WBQ Design

The traditional closed-ended WBQs, structured with qualitative categorical or dichotomous questions, seem to be advantageous psychometric attempts and are desirable options for participation, contrary to open-ended questions requiring written answers.

The WBQ of this study primarily consisted of binary questions for demographics such as gender, age, and, according to the CDC, certain underlying medical conditions that potentially contribute to more severe illness, in the case of COVID-19 [2]. The work-related questions were based on the three-sector model of classical economy, with a further tertiary sector division into critical Greek services. Concerning the lung-related core, 5-point Likert scale questions for frequency of cough, dyspnea, and sputum were included. Cold-related questions for sniffle, fever, flu, and headache were also present in our questionnaire. Further data for geographical environments, exercise, nutrition, alcohol, drugs, depression, allergies, history of family pathological diseases and cancer, a prior episode of pneumonia, their perception of air pollution, and indoor air quality were recorded, to further investigate potential predictors for susceptibility to the infection. These extra variables were considered on the basis of host parameters and environmental issues that may affect the final vulnerability to infection. Investigation for COVID-19 infection was treated as a three-categorical variable, assigning the following values: (i) identified COVID-19 infection; (ii) COVID-like/flu (CLF) symptoms; (iii) no infection/symptoms.

Considering the qualitative WBQs’ type for a better e-sample response, in addition to the fact that European countries are mainly aging, the concept of age-related questions was to follow a generation-based model with age ranges, to reveal future health-related tendencies. Generation categories included (i) baby boomers (age range 57–75 in 2021); (ii) generation X (age range 41–56 in 2021); (iii) millennials (age range 25–40 in 2021); (iv) generation Z restricted in adults (18–24 in 2021). The exclusion criteria for WBQs were those under 18 and above 75 years of age.

### 2.3. Population-Based Sample

The survey was conducted in the Greek mainland, where these strict lockdown policies were imposed. Volos is a coastal city located at the foot of the peninsula of mountain Pelion, and the second largest municipality of central Greece, with an estimated population of 144,449. In this city, there are various geographical environments, the local economy is mainly based on services, and the city is widely known for episodes of high levels of PMs and the adverse health effects of PM10 pollution [14]. Volos was an agricultural city with industrial areas, but in the last decades, its economy is similar to the European one, based solely on the tertiary sector services.

### 2.4. WBQ Administration

Adults were randomly invited to participate in the survey through emails, shares, and announcements on social networking sites or of the city’s central services. Informed consent was obtained from all subjects during accepting participation in the study. WBQs were submitted in Google forms, and data were saved in an excel sheet.

### 2.5. Statistical Analysis

Statistical analyses were conducted via SPSS statistics for Windows, Version 26.0. Cronbach’s alpha coefficient was applied for internal consistency reliability. Data normality was assessed with Kolmogorov–Smirnov test. Tests were two-tailed, and the level of statistical significance was established at *p* ≤ 0.05. Spearman coefficient was used to evaluate correlations between variables, and Mann–Whitney-U test and Kruskal–Wallis-H test were used for estimates of mean ranks among subgroups. Chi-square test was applied for comparisons of frequencies, and Bonferroni correction was used for comparisons between subgroups. Logistic regression analysis was considered for the investigation of predictors of vulnerability to COVID-19.

## 3. Results

### 3.1. The Distribution of Genders, Generations, and Economic Sectors

The population-based sample consisted of 1003 men (50.1%) and 997 women (49.9%). As regards demographics, there were 217 Generation Z (10.8%), 750 millennials (37.5%), 840 Generation X (42%), and 193 baby boomers (9.7%) counted in total. Table 1 describes the distribution of genders and generations among different economic sectors.

Females prevailed in categories of education, being unemployed, and undergraduates, whereas males prevailed in categories of primary, secondary, and tertiary sector’s public services, as well as freelancers. As expected, important differences were observed in generations—Generation Z consisted mainly of undergraduates (90%), and the older people were mostly retirees (70%).

### 3.2. Estimates of COVID-19 Severity Risk Factors

The percentage of certain underlying medical conditions for a likelihood of a severe COVID-19 by gender and generation are shown in Table 2.

Females showed higher rates of chronic lung diseases or other chronic diseases than males. Heart conditions and other chronic diseases were more likely to be reported from baby boomers, compared to the youth, millennials, or Generation X. Smoking was also less likely to be reported from the youth, compared to other generations, and millennials, compared to Generation X. Finally, obesity and overweight participants were less likely to be young and millennials, compared to baby boomers or Generation X.

Overall, 81.2% of the participants presented underlying medical conditions that could lead to a severe COVID-19 infection. As expected, it appears that comorbidities prevailed in the older generation (89.6%). No significant differences were revealed among economic sectors.

### 3.3. Vulnerability to Infection-Related Clinical Characteristics among Genders, Generation's and Economic Sectors

Respiratory and cold-related symptoms were more likely to exist in women, excluding sputum production. Generation Z and millennials were far more vulnerable than the older generations.

Cough and sputum production did not show any statistical difference among economic fields (*p* = 0.416 and *p* = 0.332, respectively). Dyspnea’s frequencies revealed a significant difference (*p* = 0.005) among subgroups, mainly between healthcare and unemployed (*p* = 0.018), and healthcare and retirees (*p* = 0.030). Sniffle’s frequencies revealed differences among economic subgroups (*p* < 0.001), for primary sector and undergraduates (*p* = 0.040), and mainly prevailed in education, compared to primary sector (*p* = 0.001), retirees (*p* = 0.002), secondary sector (*p* = 0.012), public services (*p* = 0.002), unemployed (*p* = 0.004), freelancers (*p* = 0.011), food services (*p* = 0.023), and private services (*p* = 0.018). Fever also showed different frequencies (*p* = 0.035), mainly in education, compared to secondary sector (*p* = 0.030). Headache frequencies varied among economic subgroups (*p* = 0.001), particularly for education and undergraduates, compared to retirees (*p* = 0.011 and *p* < 0.001, respectively). Finally, flu varied among economic subgroups (*p* = 0.034), again in education, compared to retirees (*p* = 0.024), freelancers (*p* = 0.032), and private services (*p* = 0.033). Table 3 describes the sum of positive frequencies for each symptom among economic sectors.

### 3.4. COVID-19 Prevalence in Genders, Generations, and Economic Sectors

In total, 1807 people (90.4%) stated they had never been infected with COVID-19, 75 (3.7%) were infected, and 118 people (5.9%) reported they had COVID-like/flu (CLF) symptoms but not a definite COVID-19 diagnosis. Between genders, 4.1% of men and 3.4% of women reported an infection, while 6.2% of men and 5.6% of women reported CLF symptoms, with no significant difference (*p* = 0.616). COVID-19 prevalence among generations and economic sectors is shown in Table 4.

Definite COVID-19 infection slightly prevailed in the healthcare field, especially compared to unemployed participants (6.6% vs. 1.7%, *p* = 0.012). However, identified infection and CLF symptoms in total were highly reported from undergraduates, compared to public service assistants, who rated the lowest percentage (13.3% vs. 6.2%, *p* < 0.001).

### 3.5. Investigation of Independent Predictors for Vulnerability to COVID-19 Infection

Overall, 49.8% of the participants reported an urban accommodation, 19.8% reported a prior episode of pneumonia, and 44.6% reported not to exercise (<day/week), with no differences among genders and generations. Moreover, 60.2% reported a stress/anxiety existence—mainly in younger generations—and 62.8% reported exposure to air pollution, with no significant relationship with COVID-19 self-reported individuals.

A final logistic regression model-fitted analysis, considering identified COVID-19 as a dependent variable, found that common headache (OR 2; CI 1189–3013; *p* = 0.007) and prior pneumonia (OR 1.9; CI 1024–2898; *p* = 0.04) were significant predictors for susceptibility to SARS-CoV-2 infection. The known risk factors were considered as a sum since they admittedly influence the overall individual’s immunity and susceptibility, and they are already known as risk factors for likely severe cases of the disease (Table 5).

## 4. Discussion

A year after the COVID-19 pandemic, the evidence indicates certain underlying medical conditions are related to severe cases of SARS-CoV-2 infection [2]. Instead of counting cases at an individual level, we have demonstrated the potential severity of the pandemic at a societal level.

Female participants reported lung and other chronic diseases, except heart conditions, in higher percentages than men. As expected, the rates of preexisting medical conditions were higher for baby boomers, contrary to younger generations. However, smoking and obesity percentages were slightly the same for older people and for Generation X, revealing that comorbidities abound for Generation X, even from earlier times.

Overall, 81.2% of the study population disclosed underlying medical conditions that could lead to a severe SARS-CoV-2 infection, with nonstatistically significant differences between genders, in obesity rates, and cardiovascular diseases (CVDs). However, lung and other chronic diseases prevailed in women, whereas smoking had a male preponderance. Unexpectedly, a high percentage of the youth (56.7%) revealed the existence of medical conditions, prior to the pandemic. Obesity and other risk factors in the modern lifestyle increase the likelihood of CVDs in young individuals [15], smoking has proved to be prevalent in Greek youth [16], and generally, stress from previous years affects the existence of future disease and overall vulnerability [17].

It seems that public health awareness has proved to be a necessity, for millennials and especially the young generation, to tackle future outbreaks of infection. The physical, mental, and medical conditions of Generation Z call for vigorous attention to the discrete biomarkers of youth’s lifestyle, starting with physical condition, immunity, and environmental parameters [18]. Considering that future public health will mainly target millennials and Generation Z, as the elderly population is prominent in our current society, methods in preventive medicine need to be chiefly established since future public health issues already abound.

A 2017 Eurobarometer study found that Greeks were the heaviest smokers in the European Union, recording 37% percent of total smokers. We have reported an almost twofold aggravation in smokers, especially in the older generation. A significant percentage of smokers from Generation X and baby boomers may be at an increased risk of at least mild airflow obstruction or other related respiratory disorders. Moreover, nonsmokers have shown lower rates of respiratory symptoms, mainly in the youth generation. Since respiratory diseases and smoking have been confirmed in critical COVID-19 cases [19], and considering the World Health Organization’s (WHO) warnings for COPD as the future third cause of deaths worldwide [20], urgent awareness should be raised so as to effectively monitor smokers, identify COPD patients or patients with underlying respiratory diseases in the primary care setting, and thus maintain public health.

Future vulnerability and lung diseases may be increased due to various factors, in comparison with the current rates of median ages [21]. We evaluated respiratory symptoms and vulnerability to infections among classical sectors of the economy with further distribution of the tertiary sector, retirees, unemployed, and university habitat, to determine vulnerability in different fields during the pandemic. However, the higher percentages that our study revealed in the unwaged category may be correlated with a weak immune system due to unemployment’s stress and depression [22]. The education field seems to be a vulnerable sector [23], and accordingly, it seems important to monitor universities, since young adults over-reported a general vulnerability, thus being prone to the current and future pathogens’ airborne transmission.

Even if droplets and aerosols can be controlled in the primary sector, naturally due to the land or sea distances, and secondary sector industries seem to be closed structures, in the tertiary sector—the sector of services, communications, and interactions—it is of paramount importance to monitor its respiratory condition, for prevention of future diffusion of infectious diseases [24]. Moreover, since in Greece, as well as other European countries, the figures of the tertiary sector surpass, at ~80%, it is required this sector be thoroughly surveilled for estimating public health maintenance policies, such as the essential use of masks in the vulnerable habitats.

Overall, 3.7% in our study reported a confirmed COVID-19 infection, with a slightly higher percentage in healthcare workers, which is increasingly indicative of airborne transmission of SARS-CoV-2 [25], while the lowest percentage was detected in unemployed participants, who exhibited a respiratory vulnerability. Yet, even self-reported identified SARS-CoV-2 infections could raise doubts since issues regarding false positives abound in all testing types [26]. However, COVID-like and cold-related symptoms but with no identified SARS-CoV-2 infection were reported almost twofold (5.9%), and mainly from the undergraduates, who could count as ground zero for a pathogen’s spread, since they are susceptible to infections and they have also revealed a general vulnerability. Considering the fact that Greece imposed a strict lockdown, and people needed an SMS authorization for basic movements, even for exercise, surprisingly, we found that Greeks are far away from being athletes, despite the fact that almost everyone was moving around with an exercise’s SMS authorization. Therefore, some fields of the tertiary sector, such as the freelancers, could be partially estimated for potential COVID-19 transmission.

Among all investigated possible predictors for potential COVID-19 infection, we found that previous pneumonia had a significant impact on the vulnerability to infection. Even if the risk for COVID-19’s severity with a history of pneumonia has recently been revealed [27], pneumonia’s postviral/postbacterial compilations, combined with other conditions, still weaken the immune system for second COVID-19 pneumonia, with either weak or severe inflammation. Since about one-fifth of the participants had reported previous pneumonia, they may be “super-spreaders” of COVID-19 if they contract the disease, as they might provide a suitable weak innate lung immunity for a viral infection, replication, and further transmission. Additionally, even if a further potential viral use of PM as carriers was not indicated, it has been proven from other studies that SARS-CoV-2 infection was correlated with air pollution [28]. Nevertheless, the specific type of PMs preferred by SARS-CoV-2 still remains unknown; further studies are needed to estimate if air pollution, smoking, or other types of PMs contribute more to SARS-CoV-2 transmission.

Vulnerability to COVID-19 inflammation is affected by various genetic factors. Most importantly, the headache was believed only to be a clinical manifestation of COVID-19 [29], but we have shown that people reporting often headaches are prone to become infected. Migraine is affected by angiotensin-converting enzymes (ACE), and it is common in people with DD allele connecting higher ACE1 activity to more headache assaults [30]. COVID-19 prevalence has negatively been correlated with ACE1 I allele frequency [31]. Thus, genetic and signal pathways of headache similar to COVID-19 pathways need to be further analyzed urgently, since COVID-19 could be a final vessels’ disease.

Thus far, no method is completely foolproof; the in toto qualitative WBQs, while not without sensitivity, appeal to the modern society to an appreciable extent, but there are concerns regarding their accuracy, contrary to the quantitative formulas, even if reliability is statistically assessed. Some citizens also tend to move to other cities due to their occupational obligations, or they are not natives, and we also have to highlight that the undergraduates spend more than half a year away from their parental city. Moreover, urbanization was not as much revealed in our study, as about half of the participants reported other geographical environments, concerning the province’s character of our study. Last but not least, e-surveillance and e-health exclude some individuals with poor engagement with social networks, and a high percentage of the older generation since social networks are advantageous mainly for younger generations. Nevertheless, Greece’s population consists of ~20% older generation, and the median age is 45.6 years; thus, our study is highly representative of age.

## 5. Conclusions

Digitized life promises impressive new insights into various health-related domains, epidemiological principles are facing challenges related to modern lifestyle, and social networks are serving as complex targets for monitoring societal conditions. We illustrated increased rates of certain comorbidities in our society, especially the heavy burden of smokers and obesity in our society, and the prevalence of cardiovascular and other conditions in the older age—as expected. Respiratory and general vulnerabilities seem higher in the education field, in both educators and trainees, in the food services, and in the unemployed individuals. Moreover, Generation Z, millennials, and specific fields in the tertiary sector services should raise awareness for future societal health issues. Moreover, we found that a prior episode of pneumonia and frequent headaches were significant predictors for a vulnerability to SARS-CoV-2 infection.

Undoubtedly, an infection’s direct widespread diffusion fluctuates among societies, related to the various physical, mental, environmental, and medical preexisting conditions, which need to be always monitored through preventive medicine’s blueprints. Yet, it is of fundamental importance to evaluate a population’s total condition and a pandemic roundly, from an individual to a societal level, with a multi-perspective point of view –the sensitivity of the epidemiological administration.

## Figures and Tables

**Table 1:** Genders and generations amongst economic sectors in Volos, *n* = 2.000.

Economic Sectors	*n*	Total Males (% Out of *n*)	Total Females (% Out of *n*)	*p*-Value	Generation Z (% Out of *n*)	Millennials (% Out of *n*)	Generation X (% Out of *n*)	Baby Boomers (% Out of *n*)
Primary	71	45 (63.4)	26 (36.6)	<0.001	2 (2.8) ^a^	29 (40.8) ^a^	34 (47.9) ^a^	6 (8.5) ^a^
Secondary	95	75 (78.9)	20 (21.1)	<0.001	3 (3.2) ^a^	35 (36.8) ^a,b^	50 (52.6) ^b^	7 (7.4) ^a,b^
Tertiary Services Public	146	87 (59.6)	59 (40.4)	<0.001	2 (1.4) ^a^	44 (30.1) ^b^	91 (62.3) ^c^	9 (6.2) ^a,b,c^
Private services	362	195 (53.9)	167 (46.1)	NS	7 (1.9) ^a^	183 (50.6) ^b^	158 (43.6) ^c^	14 (3.9) ^a^
Healthcare	106	47 (44.3)	59 (53.7)	NS	4 (3.8) ^a^	46 (43.4) ^a^	53 (50) ^a^	3 (2.8) ^a^
Food	264	143 (54.2)	121 (45.8)	NS	24 (9.1) ^a,b^	129 (48.9) ^b^	99 (37.5) ^a^	12 (4.5) ^a^
Education	146	49 (33.6)	97 (66.4)	<0.01	4 (2.7) ^a^	52 (35.6) ^b^	77 (52.7) ^b^	13 (8.9) ^a^
Freelancers	220	144 (65.5)	76 (34.5)	<0.001	7 (3.2) ^a^	87 (39.5) ^b^	107 (48.6) ^b^	19 (8.6) ^b^
Retirees	130	54 (41.5)	76 (58.5)	NS	0 (0) ^a^	2 (1.5) ^a^	37 (28.5) ^b^	91 (70) ^c^
Unemployed	287	98 (34.1)	189 (65.9)	<0.001	7 (2.4) ^a^	127 (44.3) ^b^	134 (46.7) ^b^	19 (6.6) ^b^
Undergraduates	173	66 (38.2)	107 (61.8)	<0.001	157 (90.8) ^a^	16 (9.2) ^b^	0 (0) ^c^	0 (0) ^b,c^

NS for nonsignificant. Each subscript ^a,b,c^ letter denotes a subset of age categories whose column proportions do not differ significantly from each other at the 0.05 level.

**Table 2:** Descriptive statistics for certain underlying medical conditions for a possible severe illness from COVID-19, among genders and generations in Volos, *n* = 2.000.

Common Certain Medical Conditions	Total *n* (% Out of *n*), *n* = 2000	Total Men *n* (% Out of *n*), *n* = 1003	Total Women *n* (% Out of *n*), *n* = 997	*p*-Value	Generation Z *n* (% Out of *n*) *n* = 217	Millennials *n* (% Out of *n*) *n* = 750	Generation X *n* (% Out of *n*) *n* = 840	Baby Boomers *n* (% Out of *n*) *n* = 193
Chronic Lung Diseases ^1^	220 (11.1)	93 (9.3)	127 (12.7)	0.013	21 (9.7) ^a^	78 (10.4) ^a^	83 (11.1) ^a^	28 (14.5) ^a^
Heart Conditions ^2^	358 (17.9)	168 (16.7)	190 (19.0)	0.178	26 (12.0) ^a^	99 (13.2) ^a^	149 (17.7) ^a^	73 (37.8) ^b^
Other Chronic Diseases ^3^	247 (12.4)	92 (9.2)	155 (15.2)	<0.001	17 (7.8) ^a^	82 (10.9) ^a^	106 (12.6) ^a^	42 (21.8) ^b^
Smokers	1197 (60.0)	628 (62.6)	569 (57.0)	0.011	58 (26.7) ^a^	444 (59.2) ^b^	565 (67.3) ^c^	130 (67.4) ^c^
Overweight /Obesity	770 (38.5)	394 (39.0)	376 (38.0)	0.470	57 (26.3) ^a^	265 (35.3) ^a^	359 (42.7) ^b^	89 (46.1) ^b^
**Total Risk ^4^**	1625 (81.2)	812 (81.0)	813 (81.05)	0.736	123 (56.7) ^a^	593 (79.1) ^b^	736 (87.6) ^b^	173 (89.6) ^b^

^1^: including asthma, COPD, interstitial lung diseases, pulmonary hypertension, and cystic fibrosis. ^2^: including coronary artery disease, cardiomyopathies, hypertension, and heart failure. ^3^: including chronic liver/kidney/immune/nerve diseases. ^4^: total risk refers to the sum of previous conditions, with the overlapping counted at once. Each subscript letter ^a,b,c^ denotes a subset of age categories whose column proportions do not differ significantly from each other at the 0.05 level.

**Table 3:** Descriptive statistics for vulnerability to infection-related symptoms among economic sectors in Volos, *n* = 2.000.

Sectors of Economy	*n*	Cough (% Out of *n*)	Dyspnea (% Out of *n*)	Sputum (% Out of *n*)	Sniffle (% Out of *n*)	Fever (% Out of *n*)	Headache (% Out of *n*)	Flu (% Out of *n*)
Primary	71	17 (23.9) ^a^	20 (28.2) ^a^	15 (21.1) ^a^	16 (22.5) ^a^	4 (5.6) ^a^	25 (35.2) ^a,b^	16 (25.5) ^b^
Secondary	95	19 (20) ^a^	26 (27.4) ^a^	25 (26.2) ^a^	23 (24.2) ^a^	4 (4.2) ^a^	31 (32.6) ^a,b^	16 (16.9) ^a^
Tertiary Services *Public*	146	37 (25.3) ^a^	49 (33.6) ^a^	39 (26.7) ^a^	32 (21.9) ^a^	8 (5.5) ^a^	54 (37) ^a,b^	25 (17.1) ^a^
*Private*	362	97 (26.8) ^a^	119 (32.9) ^a^	103 (27.4) ^a^	113 (31.2) ^a^	32 (8.8) ^a^	134 (37) ^a,b^	67 (18.5) ^a^
*Healthcare*	106	26 (24.5) ^a^	26 (24.5) ^a^	19 (17.9) ^a^	30 (28.2) ^a^	5 (4.7) ^a^	40 (37.8) ^a,b^	15 (14.1) ^a^
*Food*	265	72 (27.3) ^a^	73 (27.7) ^a^	67 (25.4) ^a^	81 (30.7) ^a^	24 (9.1) ^a^	108 (50.9) ^c^	54 (20.5) ^a,b^
*Education*	145	51 (32.8) ^a^	51 (34.9) ^a^	38 (26) ^a^	70 (47.9) ^b^	21 (14.4) ^a^	68 (46.5) ^b,c^	50 (34.2) ^c^
*Freelancers*	220	54 (22.7) ^a^	74 (33.6) ^a^	42 (19.1) ^a^	62 (28.2) ^a^	15 (6.8) ^a^	81 (36.8) ^a^	38 (17.3) ^a^
Retirees	130	37 (28.5) ^a^	54 (41.5) ^b^	35 (26.9) ^a^	34 (26.1) ^a^	11 (8.5) ^a^	34 (26.1) ^a^	21 (16.1) ^a^
Unemployed	287	87 (29.7) ^a^	41 (36.9) ^a^	81 (28.2) ^a^	82 (28.6) ^a^	22 (7.7) ^a^	116 (40.5) ^b,c^	54 (18.8) ^a^
Undergraduates	173	46 (26) ^a^	60 (34.7) ^a^	33 (19.1) ^a^	77 (44.6) ^b^	20 (11.6) ^a^	93 (53.7) ^c^	48 (27.8) ^b,c^

Numbers represent the sum of positive frequencies in 5-point Likert scale questions. Each subscript letter ^a,b,c^ denotes a subset of sectors’ categories whose column proportions do not differ significantly from each other at the 0.05 level.

**Table 4:** Prevalence of COVID-19 and CLF symptoms among generations and economic sectors.

Variables	Subgroups	*n*	No Identified COVID-19 Infection (% Within *n*)	Identified COVID-19 Infection (% Within *n*)	*p* Value ^1^	Total CLF Symptoms ^2^ (% Within *n*)	*p* Value ^3^
Generations	Generation Z	217	209 (96.3)	8 (3.7)	0.483	27 (12.4)	0.055
	Millennials	750	717 (95.6)	33 (4.4)		84 (11.2)	
	Generation X	840	810 (96.4)	30 (3.6)		68 (8.1)	
	Baby Boomers	193	189 (97.9)	4 (2.1)		14 (7.3)	
Economic Sectors	Primary	71	69 (97.2)	2 (2.8)	0.604	6 (8.5)	0.286
	Secondary	95	93 (97.9)	2 (2.1)		6 (6.3)	
	Tertiary Services *Public*	146	141 (96.6)	5 (3.4)		9 (6.2)	
	*Private*	362	348 (96.1)	14 (3.9)		32 (8.8)	
	*Healthcare*	106	99 (93.4)	7 (6.6)		10 (9.4)	
	*Food*	264	253 (95.8)	11 (4.2)		32 (12.1)	
	*Education*	146	138 (94.5)	8 (5.5)		17 (11.6)	
	*Freelancers*	220	211 (95.9)	9 (4.1)		26 (11.8)	
	Retirees	130	124 (95.4)	6 (4.6)		11 (8.5)	
	Unemployed	287	282 (98.3)	5 (1.7)		21 (7.3)	
	Undergraduates	173	167 (96.5)	6 (3.5)		23 (13.3)	

^1^: Chi-square comparisons of frequencies for identified COVID-19 infection and no infection. ^2^: identified COVID-19 plus CLF (COVID-like/flu) symptoms in total sum. ^3^: Chi-square comparisons of frequencies for total CLF symptoms and no symptoms.

**Table 5:** Binary logistic regression analysis with a self-reported COVID-19 identified infection as a dependent variable.

Variables	B	S.E.	Wald	df	Sig.	Exp(B)	95% C.I. for EXP(B)	
							*Lower*	*Upper*
Sum Risk factors	0.058	0.313	0.034	1	0.853	1.059	0.574	1.956
Prior pneumonia	0.544	0.265	4.197	1	0.04	1.822	1.024	2.898
Headache	0.638	0.237	7.233	1	0.007	1.993	1.189	3.01
Millennials	0.261	0.239	1.189	1	0.275	1.297	0.813	2.07

## Data Availability

Data sharing is not applicable to this article. The data are not publicly available due to restrictions. They contain information that could compromise the privacy of the participants.
